# The health status of adolescents in Ecuador and the country’s response to the need for differentiated healthcare for adolescents

**DOI:** 10.1186/s12978-017-0294-5

**Published:** 2017-02-28

**Authors:** Joar Svanemyr, Susana Guijarro, Betzabe Butron Riveros, Venkatraman Chandra-Mouli

**Affiliations:** 1 Chr. Michelsen Institute, P.O. Box 6033 Bedriftssenteret, N-5892 Bergen, Norway; 2 Adolescent Health, Standardization, Ministry of Public Health Ecuador, Av. República del Salvador 36-64, Quito, Ecuador; 3 PAHO Ecuador, Edificio de Naciones Unidas piso 8, Av Amazonas y República, Quito, Ecuador; 4 Department of Reproductive Health and Research, World Health Organization, 20 Avenue Appia, 1211 Geneva 27, Switzerland

**Keywords:** Adolescents, Youth, Differentiated health services, Sexual and reproductive health, Ecuador

## Abstract

**Background:**

Adolescents face a range of health problems but many barriers block their access to health services, and in particular to sexual and reproductive health services. The objective of this study was to assess the health needs of adolescents in Ecuador and to draw lessons from the ways the country has responded to their need for differentiated care.

**Methods:**

We conducted a literature review and consulted key stakeholders.

**Results:**

Adolescents in Ecuador today have a wide range of health care needs, in particular related to sexual and reproductive health. A major concern is the high rates of adolescent pregnancy. A national programme was established in 2007 to offer differentiated health care for adolescents—an effort that featured specially trained staff, enclaved facilities, respect for adolescents’ privacy and confidentiality, a friendly atmosphere, and a dedication to establishing trust. It resulted in rapid increases in visits by young persons both for preventive and curative services. In 2011, the government initiated a model for “integrated family and community health care” which led to a disruption of the central support for capacity building and follow-up of adolescent friendly services.

**Conclusion:**

The Ecuadorian experience has demonstrated the need for institutionalised differentiated care for adolescents who are facing a wide range of health issues.

**Electronic supplementary material:**

The online version of this article (doi:10.1186/s12978-017-0294-5) contains supplementary material, which is available to authorized users.

## Plain English summary

Adolescents face a range of health problems but many barriers block their access to health services, and in particular to sexual and reproductive health services. Adolescents in Ecuador today have a wide range of health care needs, in particular related to sexual and reproductive health. A major concern is the high rates of adolescent pregnancy. We conducted a literature review and consulted key stakeholders to assess the health needs of adolescents in Ecuador and to draw lessons from the ways the country has responded to their need for differentiated care. A national programme was established in 2007 to offer differentiated health care for adolescents—an effort that featured specially trained staff, enclaved facilities, respect for adolescents’ privacy and confidentiality, a friendly atmosphere, and a dedication to establishing trust. It resulted in rapid increases in visits by young persons both for preventive and curative services. In 2011, the government initiated a model for “integrated family and community health care” which led to a disruption of the central support for capacity building and follow-up of adolescent friendly services. The Ecuadorian experience has demonstrated the need for institutionalised differentiated care for adolescents who are facing a wide range of health issues.

## Background

Estimates and data clearly show that adolescent ill-health and death constitute a global challenge and that this age group merits special attention from the health sector [1, 2]. The health of adolescent girls, particularly their sexual and reproductive health, is of special concern for a number of reasons. These include high rates of early childbearing, related to elevated risks of complications. These young women also may be exposed to unsafe abortions, HIV infection and STIs, intimate partner violence, and sexual violence [3, 4]. In addition, mental illness, including depression, is increasing among adolescents and has been associated with other issues such as problematic perceptions of their bodies and traumatizing sexual experiences [5]. We apply the World Health Organization’s definition of adolescents as individuals in the second decades of their lives, i.e. between 10 and 19 years, and young people as individuals between 10 and 24 years.

One of the main challenges for young people is the many barriers that limit their access to health services in general, and sexual and reproductive health (SRH) services in particular. These barriers operate both on the demand and the supply sides [6–8]. On the supply side, there are issues such as cost, distance, and inconvenient opening hours. In some cases there are legal restrictions against unmarried adolescents receiving certain services and community resistance to providing adolescents with SRH information and services, for example. Adolescents may encounter health staff that have judgmental attitudes and are reluctant to provide them with SRH-related care. They also may find that the health system does not ensure the privacy and confidentiality they need. On the demand side, adolescents may not be conscious about their needs and may have a sense of invulnerability that can mean that they do not see the need to seek health services.

Adolescents in Ecuador face many of the same challenges and health problems that young people face elsewhere. The most common causes of death are suicide for girls and traffic injuries for boys [9]. Violence, including intimate-partner violence, is a major cause of morbidity and mortality for both sexes. The main issue in terms of sexual and reproductive health is the high rate of adolescent pregnancies. In Ecuador, these high rates extend to girls under 15 years of age [9].

Ecuador provides an interesting case study in adolescent health because it is in transition, and attitudes and approaches are changing [10]. Rights-based approaches have been gaining ground, and the state has acknowledged that adolescent girls and boys have rights (Art. 35, 39, 44, 45,46 de la Constitución de la República del Ecuador, 2008). It is widely accepted that adolescents need and have the right to services and information [11]. Moreover, Ecuador is a middle-income country which after 7 years of political stability is seeing increased investments in health care and declines in poverty [9]. The State increased its investments in health from US$371 million in 2004 to $1.671 billion in 2012. The current expenditure on health represents 8% of the Gross National Product (GNP), 52% of this is public spending and the rest is private [12].

In 2007, the framework of the regional Andean Plan for the Prevention of Adolescent Pregnancies was created [13]. It contributed to the establishment in Ecuador of differentiated services for adolescents—SADAs (in Spanish *Servicios para atención diferenciada de a dolescentes*). These services should have dedicated and capacitated personnel for the care of adolescents and an exclusive and adapted space according to the demand and expectations of the adolescents [11]. In 2011, however, the Government of Ecuador introduced a new policy and decided to implement a model for the provision of integrated family and community health care (in Spanish *Modelo de atencion integral del sistema nacional de salud familiar comunitario e intercultural* (MAIS-FCI)) whose key guiding principle was that integrated care must be provided throughout the lifecycle. We will return to the effect of this change in policy.

### Scope and objectives of the study

This article seeks to describe the health care needs of adolescents in Ecuador today with a focus on sexual and reproductive health. It shows these needs have and are being met within the current health system of Ecuador and reviews the implications of recent changes in the organization of health services, from dedicated to integrated care, for adolescents.

The objective is to draw lessons from Ecuador’s approach and to inform policy makers, programme managers, international organizations, and other stakeholders on how to provide adolescent-friendly care—care that is differentiated and includes a focus on sexual and reproductive health.

## Method

The authors searched for relevant literature in Pubmed, Popline, and Google Scholar, and among reference lists of papers identified as relevant. The search terms used were different combinations of the words *health, health care, health services, sexual and reproductive health, adolescents, youth, young people,* and *Ecuador*. The search included all publications made before 1^st^ of November 2014. They also consulted—with no defined time limits applied—plans, reports, and other grey literature published by a range of organizations and institutions, including the Ministry of Health of Ecuador, UNFPA, and UNICEF. We included all the publications (journal articles and grey literature) that met the following criteria: descriptions and/or analysis of health behaviors and problems of adolescents in Ecuador; response of the health system to adolescents in Ecuador; published in English or Spanish; and published before 1 November 2014. Given the dearth of publications on adolescent health in the country this, we used all the publications available. Literature that did not include data from Ecuador and on adolescents/youth/young people was excluded.

Complementary information was sought through informal consultations with key stakeholders and health personnel in Ecuador in October 2014. In one meeting the first and second authors met with representatives of UNFPA, UNICEF, the Ministry of Public Health, the Ministry of Social Welfare of Ecuador, four nongovernmental organizations (NGOs), and two youth representatives. Subsequently they met with the staff of three Ecuadorian health centres—one in Quito and two in rural areas—and one hospital. The representatives were selected based on their knowledge with the country’s health policy and health system and/or familiarity with adolescents’ health. The discussions evolved around the present status of adolescents’ health, adolescent’s challenges related to SRH, and the health sector’s response to these issues.

## Results

### Health status of adolescents in Ecuador and adolescents’ need for health services

#### Traffic injuries, suicide, and violence

The most common cause of death in 2011 among adolescent girls was suicide (13% of all deaths), as opposed to traffic injuries among adolescent boys (19%) [9]. Traffic injuries were the second most common cause of death for girls (8%). For boys, violence was the second most common cause (12%), and suicide the third (7%).

#### Mental health and suicide

There is a growing recognition worldwide that mental ill health among adolescents has been a neglected issue [5]. The indicators for Ecuador are definitely a matter of concern. The country is among those with the highest suicide rates among young people in the Americas region. Further, these rates have been increasing [14]. Rates are higher for women than for men in the age group 10–19 (7.1% vs 6.4%), but higher for men in the age group 15–24 (13.0% vs 9.3%). A 2007 school-based health survey among students aged 13 to 15 years found that 17% in the cities of Guayaquil, 17.5% in Quito, and 19.4% in Zamora had considered attempting suicide during the preceding 12 months [15].

#### Violence

Another major national concern is Ecuador’s high level of violence among adolescents. The school survey quoted above found that 34.8% of students in Guayaquil, 36.2% in Quito, and 36.1% in Zamora had been physically attacked one or more times during the preceding 12 months. Girls and women also have a high risk of being exposed to violence by their partners. The lifetime prevalence of intimate partner violence (IPV) is estimated at 35% for physical violence, 14.5% for sexual violence, and 43.4% for psychological violence (INEC 2011 quoted in [16]).

#### Alcohol use

Alcohol consumption often starts early among adolescents in Ecuador. A national survey of drug use among students in 2008 found that the mean age for trying alcohol for the first time was 12.8 years [17]. In 2014, the Ministry of Public Health reported that 45.6% of the Ecuadorian population (47.2% of men and 43.9% of women) between 10 and 19 years, and 21.3% of children between 10 and 14 years declared that they had tried alcohol. Some 12.9% of 10–14-year-olds reported having been drunk at least once. As many as 24% of adolescents 13 to 15 years old in Guayaquil, 27.7% in Quito, and 27.8% in Zamora, said they had consumed so much alcohol that they had been “really” drunk one or more times in their lives [15]. Having tried alcohol early is more common among adolescents in indigenous groups and in the poorest quintile of the population [18].

#### Overweight and obesity

The 2007 school-based survey also found that 28.6% of the 13- to 15-year-old students in Quito were overweight and 7.3% were obese [15]. A study of a group of urban and rural Ecuadorian adolescents reported that dyslipidemia, abdominal obesity, and overweight conditions were prevalent in 34.2, 19.7, and 18.0%, respectively, of the population and that 59% of Ecuadorian adolescents have poor levels of physical fitness [19]. Just over a fourth of Ecuadorians between 10 and 18 years old exceeded the recommended minimum level of physical activity [18].

#### Early child bearing and adolescent maternal health

The major national issue concerning adolescents’ SRH is the high rate of teenage pregnancies. The National Demographic and Maternal and Child Health Survey (ENDEMAIN) in 2004 found that more than 20% of Ecuadorian women surveyed (age 15–49) had had a child or had been pregnant when they were adolescents [20]. Early pregnancies carry elevated risks of complications both for mother and the child, and are considered an obstacle to the social and economic development of families and communities [21, 22]. Some 4% of deaths among female adolescents in Ecuador are due to complications during pregnancy and birth [9].

According to 2010 statistics from National Statistics and Census Institute (Instituto Nacional de Estadística y Censos—INEC), 44.1% of mothers had their first child when they were 15 to 17 years old, and 2.4% had their first child when they were between 12 and 14 years old. Some 3.4% of the approximately 3.6 million mothers in Ecuador in 2010 were between 12 and 19 years old, which corresponds to a reported figure of 122 301 adolescent mothers [20]. The percentage of adolescents in the age group 15–19 with a least one child increased from 14.84% in 1990 to 17.53 in 2010 [20]. Data from 2013 on births show that 19.48% of all births corresponded to women aged 15 to 19, and 0.76% corresponded to girls under the age of 15 [20].

#### Child bearing, socioeconomic level, and school attendance

In 2012, some 84% of Ecuadorians between 15 and 17 years old went to school, but three out of 10 were lagging behind in their studies—that is, not attending the grade levels corresponding to their ages. Of these, half were from indigenous communities [20]. The proportion of mothers younger than 19 years is highest in groups with low income and low education. Forty-seven percent of adolescents who were of low socioeconomic status became mothers or were pregnant during adolescence. About 70% of Ecuadorian mothers who became pregnant during adolescence already had quit school before becoming pregnant. Of the remaining 30%, some 12% continued their studies, whereas 18% interrupted their studies because of the pregnancy. Apparently 70% of those who interrupted their studies did not return to school [20].

#### Sexual activity and contraceptive use

As can be expected, the high number of pregnant adolescents is due to a combination of early sexual activity and low or inadequate use of contraception. The ENSANUT survey in 2012 found that almost six of 10 women between 15 and 24 years old had had a sexual experience. Some 54.6% of them had had this experience before they were married. This represents a considerable increase from 2004, when only 46.7% of those aged 15 to 24 had had a sexual experience, and 37.2% reported having had a sexual experience before marriage [20]. The school-based survey quoted above found that 26.0% of boys and 7.1% of girls aged 13 to 15 in Guayaquil had had sexual intercourse at least once [15]. The corresponding figures were 23.4% of boys and 8.1% of girls in Quito and 33.7% of boys and 9.9% of girls in Zamora 13 to 15 years had had sexual intercourse at least once.

According to the ENDEMAIN study in 2004, almost all adolescents (97%) knew about modern contraception methods; 47% were using them at the time of the survey, and 13.5% used them during their first sexual relationship (22). At the time of the ESANUT 2012 survey, the percentage of adolescents (15 to 19 years) reporting the use of contraception had increased to 68.9% [23]. Overall, the use of modern contraception has almost tripled over the last two decades among adolescents [23]. A national study found that 89% of those who were sexually active before they were 17 years old had not used a condom during their first sexual encounter (González-Rozada, 2010 quoted in MCDS 2014).

A study conducted in six secondary schools in the city of Cuenca, Ecuador, and in 20 secondary schools in Cochabamba in Bolivia found that sexually active adolescents (aged 14 to 18) who consider gender equality as important reported higher current use of contraceptives within the couples they had formed. They also were more likely to describe their last sexual intercourse as a positive experience, and considered it easier to talk with their partners about sexuality, in comparison to sexually experienced adolescents who were less positively inclined towards issues of gender equality [24].

#### Sexual violence

Research shows that a relatively high number of sexual relations result from violence or are accepted under circumstances where one of the partners, normally the girl, does not feel she can refuse [25]. Some 14.6% of the Ecuadorian women aged 15 to 49 reported in 2004 that they had experienced sexual violence in their lifetimes [25]. Less than a third (27.6%) of the students in a high school survey conducted in Rumiñahui County in the Province of Pichincha believed that it was acceptable to refuse sex at any time, and less than a quarter (23.4%) of students believed that it was not acceptable to refuse to sex under any circumstance, whereas half (49.0%) believed that the right of refusal depended on the situation [26].

#### Association between gender-based violence and early pregnancy

There seems to be an association between gender-based violence and pregnancy before age 18. Intimate partner violence is more common among those who become pregnant in early adolescence than it is among those who do so later. According to a governmental report published in 2014, women who became pregnant during adolescence are between 1.55 and 1.66 times more likely to be abused physically or psychologically by their partners or ex-partners than women who did not experience pregnancies during adolescence [20]. Having suffered from sexual abuse during childhood or adolescence was found to be a risk factor for adolescent pregnancy in a study conducted in Ecuador’s Amazon basin [27]. Likewise, a hospital-based study in Quito found that sexual abuse was more than three times as common among pregnant adolescents as compared to adolescent women who were not pregnant (14.9% vs 4.5%) [28].

#### Indigenous groups are more vulnerable

Some 9% of Ecuadorians from 0 to17 years old belong to indigenous groups, and 8% belong to Afro-Ecuadorian groups [9]. Adolescents from these groups, tend to score lower for all indicators of health, education, access to services, and poverty. For example, members of these groups have higher school drop-out rates, higher rates of teenage pregnancy and sexually transmitted infections (STIs), including HIV, lower rates of skilled medical care during child delivery, and higher rates of tobacco and alcohol use [29]. Some 66.7% of adolescent girls aged 15 to 19 in the Amazonian province of Orellana were pregnant at the time of the 2004 survey, as compared to 13.9% of adolescents in the most populous province of Guayas (ENDEMAIN 2004 quoted in Cabrero et al., 2010).

#### Gender issues

Gender inequality, for both girls and boys, is intensified by cultural norms (the “machismo”) that expects boys to be aggressive and dominant and girls to be submissive and obedient. This limits their ability to protect themselves and leaves them vulnerable to a wide range of health risks.

What we may conclude from the data presented above is that adolescents in Ecuador have a high risk of being exposed to violence, have worrying levels of mental illness, and are vulnerable to alcohol abuse, obesity, and insufficient physical activity. In addition, there is a pattern of early sexual debut with a lack of or limited knowledge on how to protect themselves and prevent unintended pregnancies, leading to increased adolescent fertility. International research has demonstrated that these issues often are connected. For example, alcohol use and intimate partner violence tend to be associated with early sexual debut and low use of contraception [30]. Research on female suicide has suggested that intimate partner violence and adolescent pregnancy are risk factors and that females who commit suicide are more likely to have experienced sexual abuse [14]. The situation of adolescents in Ecuador underscores the need to reach out to them with health information and to make health services youth-friendly and hence attractive to this age group.

### Use of health services by adolescents in Ecuador

**Fig. 1:**
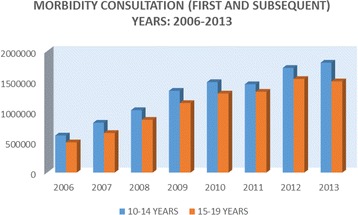
Consultations for morbidity (first and subsequent)

**Fig. 2:**
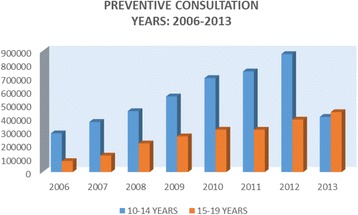
Preventive consultations (first and subsequent)

Since 2007, the Government of Ecuador has worked to make access to health care free of charge and there has been a considerable increase in the use of health services. Data relating specifically to adolescents’ use of health services is somewhat limited. There are, however, statistics on the number of adolescents in the age groups 10–14 and 15–19 who came to health facilities for preventive and curative services. As can be seen in Figs. 1 and 2, the numbers have increased steadily.

For more service-specific information, skilled attendance during delivery is one of the few indicators for which data is available. In recent years, young women have been more frequently attended by skilled personnel during child birth. As noted earlier, pregnancy, delivery, and postpartum conditions are by far the most common reasons for the hospitalization of adolescents (10 to 17 years old); some 50% of adolescent hospitalizations are related to this [9].

#### Sources of information

Adolescents have limited access to information and to adult-led teaching on health matters. A survey conducted in seven high schools in 2005 in the province of Pichincha found that the most important source for general information about SRH was the media (83% of respondents). Only 14% reported that they received information from school. 60.9% responded that they had conversed with their parents about SRH, but only 11% cited their parents as information sources. Lack of trust and embarrassment were given as the main reasons for not discussing SRH with parents. Two percent reported obtaining SRH information from their friends. When it came to information, more specifically about family planning, school was the main source of information (23%) [26]. While only a small proportion of adolescents reported receiving information on SRH from their parents, a national UNFPA survey on perceptions surrounding family planning and contraception found that a large majority of the population is in favour of giving adolescents access to contraception, and in favour of providing adolescents with information about its use [31].

#### Barriers to the use of SRH services and access to information (structural, economic, socio-cultural)

Adolescents and young people face a wide range of barriers that can limit their access to health services. A study conducted on behalf of the Regional Andean Committee for the Prevention of Adolescent Pregnancies, divided the barriers of access into three main groups: sociocultural, institutional, and political (national or territorial) [7]. The last group includes legal and regulatory barriers. Low availability of health services, high costs, low quality, and lack of resources—including human resources—are some of the main barriers on the supply side.

Adolescence is commonly seen in Ecuador as a period prone to risks and problems, especially in matters of sexuality. Even though the UNFPA data presented above indicate that many people accept that adolescents should be able to access contraception, adolescents often encounter strict norms and moralistic attitudes from adults [9]. Gender relations are characterized by machismo (sexism), homophobia, and the expectation that women should become mothers early. Instead of providing information and encouraging/supporting their daughters to protect themselves from sexual and reproductive health problems, parents and guardians tend to try to prevent young women from having contact with men [9]. For many Ecuadorians, the only acceptable way for young women to avoid pregnancy is for them “not to go with men”. The national study of 2011 found that 68% of women and 61% of men did not approve of sexual relationships among adolescents [31]. These attitudes also are common among health workers [7, 32, 33]. Given this, one of the main barriers to adolescent access to contraception—and a major reason for the gap between knowledge about contraception and its actual use—is the attitude of health workers who believe that contraception is not an adequate response to sexual activity among adolescents [20].

At the same time, maternity is the natural horizon for many young women, and in particular for those from poorer regions, as motherhood allows them to be materially and symbolically acknowledged by their families, communities, and the State. Maternity also gives young women rights and services guaranteed by the Government [20]. Studies have found that many adolescents girls believe that for them there are no good alternatives to early motherhood for being acknowledged as adults and as responsible persons [7, 34]. This confluence between limited access to services and information on sexual and reproductive health on the one hand, and the idealization of motherhood on the other, leaves adolescents vulnerable not only to early pregnancies but also to sexually transmitted infections and to sexual violence.

#### Introduction and scaling up of differentiated health care for young people (2007–2011)

The following section describes the development in Ecuador of differentiated health services for adolescents. This differentiated approach was conceived to respond to the challenges and needs described above. In 1988, Ecuador saw the start of a historic process with the opening of a ‘Servicio de atencion integral para adolescentes embarazadas’ (Service for the integrated care of pregnancy adolescents) at the Isidro Ayora de Quito Obstetrics-Gynaecology Hospital. The establishment of this service made visible the demand and the need for health services among adolescents. In 1992, the National Programme for Adolescent Health developed a manual of norms and procedures for comprehensive adolescent health care. The manual was developed by medical professionals and employed a biomedical approach [10]. In 2005, the National Policy for Sexual and Reproductive Health identified adolescent pregnancies as a priority issue and stated that services for them must be strengthened [35]. However, until 2007, only three public health units other than the Isidro Ayora hospital, were in place to provide differentiated care for adolescents [7]. The adolescent friendly services at Hospital Isidro Ayora in Quito, as well as three primary level units, were initiated and continued on the initiative of individuals and groups particularly interested in the addressing the health problems of adolescent. The model was not expanded nor replicated until 2007 mainly because of lack of political will.

In 2007, the Andean Plan and the National Plan for the Prevention of Adolescent Pregnancy [13] and in 2009, the New Guidelines for Comprehensive Care of Adolescent Health, were launched [11]. The National Plan for Adolescent Pregnancy Prevention uses a rights-based approach and is founded on the assumption that for adolescents to exercise their reproductive rights they not only need access to a network of services but also must be empowered to take control of their sexuality. A primary issue addressed by the plan was the urgent need for information on vulnerable or marginalized groups requiring priority attention. These included young adolescents (10–14 years); adolescents with little education; those out of school; rural and indigenous groups; those living in isolated areas of the country; as well as migrants, internally displaced persons, and refugees.

The 2009 Guidelines for Comprehensive Care for Adolescent Health promoted care based on: (i) differentiated services delivered with a comprehensive, intercultural, participatory, and rights-based approach; and (ii) friendly care characterized by respect, confidentiality, positive attitudes among health care workers, and appropriate skills and competencies among such workers. The model of differentiated health care was named Servicios de Atención Diferenciada para Adolescentes (SADA). This new focus aimed to facilitate access of adolescents (10–19 years old) to health services. The Ministry of Public Health (Ministerio de Salud Pública—MSP) promoted the implementation of differentiated services for the integrated care of adolescents in first-level health units and in hospitals based on a “normative package” for such care (norms, protocols, and quality standards) and on sensitization of and capacity building of the multi-disciplinary personnel in health establishments. The guidelines referred to above state that differentiated services should include “trained full time personnel who provide care grounded in rights-based, gender-sensitive, culturally-sensitive, participative and integrated approach”, and that they should do this in a “placed adapted to the needs and preferences of adolescents, and assigned to their use only” [11].

To address the problem of early pregnancies, the Government formulated the National Inter-sectoral Strategy for Family Planning and the Prevention of Adolescent Pregnancies (in Spanish *Estrategia Nacional Intersectorial de Planificación Familiar y Prevención de Embarazo en Adolescentes* (ENIPLA) in 2011. This framework called for action in four areas:1) maintaining adolescents in the education system and strengthening comprehensive sex education, 2) improving adolescents’ access to SRH services including methods of contraception, 3) family and community action, social dialogue and co-responsibility, and 4) promoting changes in sociocultural patterns.

Also in 2011, a major shift in policy was announced as the Ministry of Public Health launched a model for the provision of integrated family and community health care (*Modelo de atención integral del sistema nacional de salud familiar comunitario e intercultural* (MAIS-FCI)). MAIS conceptualizes bio-psycho-social quality care with an emphasis on prevention and promotion that pays attention to every person in an integrated way and supports human development. The MAIS framework gives priority to integrated care throughout the lifecycle and to family and community medicine, which, by consequence, meant the abolition of vertical programmes. As can be expected, this had an effect on the differentiated approach taken for adolescents. At the national level, the adolescent programme was dis-established, and at local level, the transition process was affected by a lack of guidance from the new model in terms of management of the SADAs. However, in the National Plan for Wellbeing (2013–2017) there is a call to guarantee effective access to integrated services for sexual and reproductive health [9].

#### Achievements of the differentiated services programme

A survey was carried out by the Ministry of Public Health in collaboration with UNFPA to map the status and the achievements of the SADAs, in 2012 [36]. Responses were received from 74 of the 158 SADAs which reports suggested were in place in 2011 (a 46.8% response rate), and from 14 of 24 provinces (a response rate of 58%). In addition, responses were received from 26 new health facilities that had initiated SADAs during the year before the survey, bringing the total number of SADAs that responded to 100 (out of the composite total number of 184). Some of the SADAs which did not respond had allegedly stopped offering differentiated services for adolescents due to staff shortages or reorganizations.

The survey results showed that the number of SADAs gradually increased from 38 in 2008 to 97 in 2009, to 139 in 2010, to 158 in 2011. In 2011, all 24 provinces had differentiated care for adolescents. There were only two to three SADAs in each of five provinces and four SADAs in each of nine other provinces. Further, there were 27 SADAs in Guayas, 29 in Pichincha, and 15 in Manabi. This means that these three most populous provinces, which together had 52.5% of the country’s population in 2010, had 38.5% of the SADAs in 2012 [36].

Of the 14 responding provinces, 11 reported significant increases in service use by adolescents, and two reported a decrease (Pichincha, down 30%, and Cotopaxi, down 9%). From 2008 to 2011, the number of prevention services offered increased in these 100 units from 138,787 visits to 264,924, this represented a 90.9% increase. A large part (57.5%) of this increase, however, occurred in one province: Guayas, which has 25% of the nation’s population, reporting 72,521 more consultancies in 2011 (from 63,792 visits to 136,313). This means that for the rest of the country, the increase was 71.5% (53,616 more consultancies, up from 74,995 to 128,611). The trend was the same for the two other types of services covered by the survey (consultancies for morbidity and pregnancy) [36].

The survey found that in 2011, 1061 health professionals were sensitized and trained to attend to adolescents in these 100 units. A third (32%) were medical doctors; 20% were obstetricians; 14% were nurses or odontologists; 4% were psychologists; and 2% were social workers and nutritionists [36].

In terms of community sensitization about the SADAs and the need to prevent adolescent pregnancies, 63,004 persons were reported to have attended educational activities (almost one third of them in Guayas). These activities were offered to adolescents (in 83% of the cases), to fathers and mothers (11%), to teachers (2%), and to others such as police officers, firemen, civil defence officials, and community leaders (4%) [36]. The survey did not collect data on the extent that disadvantaged groups benefitted.

The elements and achievements described above preceded the institutionalization of integrated care for adolescents within the first level of MAIS-FCI. The rationale for this new approach, as stated above, was to reduce tendencies towards the fragmentation and segmentation of services and systems in a way that might compromise the quality of service provision and lead to inequalities in access to services. This meant a move away from differentiated care for particular groups towards an approach in which all groups—in theory—would receive the same level of care throughout their lifecycles.

Consequently, the capacity building, the support systems and the follow-up for the SADAs were discontinued. It also meant that local health authorities in many cases no longer considered adolescents as a group that should receive differentiated care. That fairly quickly led to a situation wherein staff who had been trained to care for adolescents felt ignored and rudderless, and found themselves no longer in a position where they could use the skills for which they had been trained [36]. Interviews with health personnel who had been trained to provide differentiated services for adolescents indicated that they subsequently had to work with all groups of patients and were confused about their roles [36].

Consultations held with health personnel in the context of this study confirmed that some of the problems caused by the transition from SADAs to MAIS, were: uncertainty related to the competencies needed to serve the broader population; lack of technical skills; discontinuation at the central level of management and follow-up of training and implementation; loss of data collected by the SADAs; and dismantling of the SADAs by local authorities. Reportedly, some facilities in some places continue to offer differentiated care for adolescents within the MAIS framework while also extending services to the population as a whole. However, at times this continued differentiated care takes place under difficult conditions, such as the loss of dedicated time slots and/or work spaces. Continuation of adolescent-targeted care now may depend to a large degree on the presence of committed persons and on local management willing to assign young people priority.

Meanwhile, data on the uptake of services and on how the transition from the previous model has been perceived by adolescents are scarce. One important reason for this is that reports are no longer collected from the information system previously established by the SADAs.

## Discussion

Findings from this study highlight the pressing need for differentiated, youth-friendly health care in Ecuador. Adolescents are vulnerable to a range of health problems, including early and unwanted pregnancy, intimate-partner violence, alcohol and drug abuse, depression, and suicide. Yet they have limited access to adequate preventive, curative and rehabilitative services. Some of the most important barriers are sociocultural and attitudinal, among both the general population and among health workers. These barriers have led to poor demand among adolescents for SRH services.

The case of the SADA model in Ecuador offers some interesting lessons. First, it demonstrated that there was an unmet demand for youth-friendly differentiated services. Second, it showed that it was possible to meet this demand by offering differentiated care that paid attention to the specific needs of adolescents. The approach was effective, at least in terms of increasing the uptake of health services, in part because it was friendly, respected confidentiality, and built confidence among persons of this age group. Over 3 years, the number of SADAs increased from 38 to 158. National coverage was accomplished—in the end, there were SADAs in all of Ecuador’s 24 provinces, which was followed by a considerable increase in the number of adolescents coming for preventive services and when injured or ill. These young people also came for antenatal and delivery care services. The 100 SADAs responding to the 2012 survey reported almost a doubling of the number of adolescents seeking preventive services between 2008 and 2011 [36].

The subsequent shift in Ecuador from a health model targeting various periods of the lifecycle with specific programmes to a model of integrated care demonstrated the fragility of the achievements. When the institutional structure and the support system for adolescents changed, the staff trained to offer differentiated care were no longer, in many cases, able to devote attention on adolescents. Goicolea et. al., who studied three SADAs in 2010, foresaw this situation when they observed that the sustainability of the youth-friendly services was threatened by a lack of funding and clear enabling structures in terms of training and close support [10].

Further, following the changes, because health facilities were not required to report on adolescent-specific activities and services, the Ministry of Public Health has not collected data that would enable an assessment of the extent of subsequent use by adolescents of health services, or an assessment of whether adolescents are satisfied with the services offered under the current integral MAIS model. As also observed by Goicolea et. al. (2012), the authors of the current report note a recent lack of accurate data, combined with inconsistent routines for registering data. The lack of monitoring and evaluation of the differentiated services offered to youth in Ecuador reflects the situation in many countries [6, 8]. Consequently, within the current system, the specific needs of adolescents are to a large extent, not visible and not recognized. This carries the risk that the responses to their needs and problems are not adequate.

## Conclusion

This study clearly confirms what has been observed by others: adolescents need access to quality, adolescent-friendly services provided by clinicians trained to work with this population group. Service providers must be trained so that they understand the needs of adolescents and youth, know how to communicate with and counsel them effectively, and know how to provide services in supportive, non-threatening ways [37]. Ongoing training and support are essential for health providers to feel secure enough to implement a service that may be unfamiliar to them [10]. Strategies for adolescent-friendly services must be tailored to the developmental needs of this age group and to the social context, and must employ multifaceted approaches. Further continued investment in effective prevention and treatment strategies is essential [4]. As WHO has noted, for health services to be youth friendly, they need to be available, accessible, acceptable, and equitably offered to different youth sub-populations [38].

The study also highlights the need for accountability mechanisms, rigorous monitoring systems, and indicators related to adolescent access to health services. This information must extend beyond figures on skilled attendance at delivery and unmet needs for contraception.

Despite increasing investment and an overall improvement in health services in Ecuador, there is a pressing need to step up efforts to provide effective care to adolescents. Barriers that currently limit the provision of such care and restrict its use must be eliminated. Many adolescents do not feel welcome or comfortable in seeking health services that—in their perception—do not always guarantee confidentiality. Among other things, barriers affect access to and use of SRH services and contraception. The best approach appears to be to take steps to guarantee differentiated, integrated care to adolescents. Such health services should reflect adolescents’ needs for confidentiality and privacy, and should be offered in a manner that is friendly and inclusive. Within the strategy for promoting family planning, the Ministry of Public Health has sought to improve access to free contraception and to provide health personnel with the tools and skills needed so that they can provide contraceptive counselling. However, it is a challenge for Ecuador to respond in an integrated manner to the problems of early pregnancy, mental health, and violence, which currently occur at high rates among adolescents. Another challenging task is to maintain differentiated care in an environment of restructuring towards a model of services for all population segments. Despite good intentions, the recently adopted model led to the disruption of important elements of the pre-existing programme for differentiated health care for adolescents, including centrally led capacity building and support functions.

Ecuador is a country in demographic transition. To improve opportunities for adolescents and youth, a policy that considers their specific needs for health, education and economic development, must be developed and implemented. Such a policy must call both for inter-sectoral efforts and for the participation of young people. In health system transition processes, such as the one that Ecuador has undergone, strategies to overcome barriers to the provision and use of health services by adolescents must be safeguarded. It is important to maintain the competencies and morale of staff who have been trained for adolescent care through ongoing training and support, and to inform these health workers effectively about changes in the system so that they can continue to offer quality care. Norms and standards that allow and enable monitoring and feedback are also essential, as is the maintenance of channels of communication and the maintenance of information systems to identify good practices and identify difficulties encountered in offering integral care to adolescents.

Finally, it is necessary to introduce into the curricula of pre- and post-graduate courses of relevant disciplines (medicine, obstetrics, psychology, social work education, and law), issues related to adolescents and their rights, particularly their rights in matters of sexual and reproductive health. Comprehensive sexuality education also should be included in these curricula with the objective of ensuring that such professionals graduate with the knowledge, understanding, and skills required to provide effective services to adolescents.

## Additional file

Additional file 1: El estado de salud de adolescentes en Ecuador y la respuesta del país a la necesidad de una atención diferenciada para adolescentes. (DOCX 103 kb)

## Abbreviations

ENDEMAIN: The National Demographic and Maternal and Child Health Survey
ENIPLA: Estrategia Nacional Intersectorial de Planificación Familiar y Prevención de Embarazo en Adolescentes (National Intersectorial Strategy for Family Plannning and Prevention of Adolescent Pregnancy)
HIV: Human Immunodeficiency Virus
INEC: Instituto Nacional de Estadística y Censos (National Institute for Statistics and Censuses)
MAIS-FCI: Modelo de atencion integral del sistema nacional de salud familiar comunitario e intercultural (Model for integral care of the national system for comunal family and inter-cultural Health)
MCDS: Ministerio Coordinador de Desarrollo Social (Ministery for Coordination of Social Development)
MSP: Ministerio de Salud Pública (Ministry for Public Health)
NGO: Non-Governmental Organization
SADA: *Servicios para Atención Diferenciada de Adolescentes* (Services for differentiated care for adolescents)
SRH: Sexual and Reproductive Health
STI: Sexually Transmitted Infection
UNFPA: United Nations Population Fund
UNICEF: United Nations Children’s Fund
WHO: World Health Organization
