# Supplementary material for: The health status of adolescents in Ecuador and the country’s response to the need for differentiated healthcare for adolescents
Source: Reprod Health. 2017 Feb 28;14:29. doi: 10.1186/s12978-017-0294-5 (PMC5331697; doi:10.1186/s12978-017-0294-5)
Supplement: Additional file 1: — El estado de salud de adolescentes en Ecuador y la respuesta del país a la necesidad de una atención diferenciada para adolescentes. (DOCX 103 kb) [file 12978_2017_294_MOESM1_ESM.doc]

**El estado de salud de adolescentes en Ecuador y la respuesta del país a la necesidad de una atención diferenciada para adolescentes.**

Joar Svanemyr1, Susana Guijarro2, Betzabe Butron Riveros3, Venkatraman Chandra-Mouli4

Afiliación institucional:

1. Chr. Michelsen Institute, P.O. Box 6033, N-5892 Bergen, Noruega.

2. Salud del Adolescente. Normatización. Ministerio de Salud Pública Ecuador, Av. República del Salvador 36-64. Quito, Ecuador.

3. OPS Ecuador, Edificio de Naciones Unidas piso 8, Av. Amazonas y República, Quito, Ecuador.

4. Departamento de Salud Reproductiva e Investigación, Organización Mundial de la Salud, 20 Avenue Appia, 1211 Ginebra 27, Suiza.

Autor correspondiente:

Joar Svanemyr.

Chr. Michelsen Institute

CORREOS. Box 6033 Bedriftssenteret

N-5892 Bergen, Noruega

Teléfono: (+47) 47938071

E-mail: [joars@cmi.no](mailto:joars@cmi.no)

**Resumen**
**Antecedentes**: Los adolescentes se enfrentan a una serie de problemas de salud, y muchas barreras dificultan su acceso a los servicios de salud, en particular, a los servicios de salud sexual y reproductiva.

**Objetivos**: Evaluar las necesidades de salud de los adolescentes en Ecuador y obtener lecciones de la respuesta del país a la necesidad de atención diferenciada.

**Métodos**: Se realizó una revisión de la literatura y se consultó con actores clave.

**Resultados**/**discusión**: Actualmente, los adolescentes del Ecuador tienen una amplia gama de necesidades de atención de la salud, en particular aquellas relacionadas con la salud sexual y reproductiva. Una preocupación importante son las altas tasas de embarazo adolescente. En 2007 se creó un programa nacional de atención integral diferenciada de la salud de adolescentes, un esfuerzo que incluyó personal especialmente capacitado, instalaciones específicas, respeto a la intimidad y confidencialidad de adolescentes, y un ambiente amigable y confiable. El resultado fue un aumento rápido de la demanda de adolescentes y jóvenes tanto para los servicios preventivos como para los curativos. En 2011, el gobierno inició un modelo de "atención integral de la salud familiar y comunitaria", lo que provocó una interrupción del apoyo central para la creación de capacidad y el seguimiento de los servicios amigables de los adolescentes.

Conclusión: La experiencia ecuatoriana ha demostrado la necesidad de institucionalizar la atención integral y diferenciada para adolescentes quienes enfrentan una amplia gama de problemas de salud.

**Plain English summary**

Adolescents face a range of health problems but many barriers block their access to health services, and in particular to sexual and reproductive health services. Adolescents in Ecuador today have a wide range of health care needs, in particular related to sexual and reproductive health. A major concern is the high rates of adolescent pregnancy. We conducted a literature review and consulted key stakeholders to assess the health needs of adolescents in Ecuador and to draw lessons from the ways the country has responded to their need for differentiated care. A national programme was established in 2007 to offer differentiated health care for adolescents – an effort that featured specially trained staff, enclaved facilities, respect for adolescents’ privacy and confidentiality, a friendly atmosphere, and a dedication to establishing trust. It resulted in rapid increases in visits by young persons both for preventive and curative services. In 2011, the government initiated a model for “integrated family and community health care” which led to a disruption of the central support for capacity building and follow-up of adolescent friendly services. The Ecuadorian experience has demonstrated the need for institutionalised differentiated care for adolescents who are facing a wide range of health issues.

Keywords: adolescentes; juventud; Servicios de salud diferenciados; Salud sexual y reproductiva; Ecuador

**Introducción**

Estimaciones y datos muestran claramente que la mala salud y la muerte de los adolescentes constituyen un desafío global y que este grupo de edad merece una atención especial del sector de la salud [1, 2]. La salud de las adolescentes, particularmente su salud sexual y reproductiva, es motivo de especial preocupación por varias razones. Estos incluyen altos índices de embarazos y parto tempranos, relacionados con elevados riesgos de complicaciones. Estas mujeres jóvenes también pueden estar expuestas a abortos inseguros, infección por VIH y las ITS, la violencia de la pareja y la violencia sexual [3, 4]. Además, la enfermedad mental, incluida la depresión, está aumentando entre adolescentes y se ha asociado con otros factores, como las percepciones problemáticas de sus cuerpos y las experiencias sexuales traumatizantes [5].

Aplicamos la definición de adolescentes y jóvenes de la Organización Mundial de la Salud, es decir, adolescentes son los individuos en la segunda década de su vida, entre 10-19 años, y jóvenes aquellos individuos entre 10-24 años.

Uno de los principales desafíos para los jóvenes son las numerosas barreras que limitan su acceso a los servicios de salud en general, y a los servicios de salud sexual y reproductiva (SSR) en particular. Estas barreras operan tanto en la demanda como en la oferta [6-8]. Por el lado de la oferta, hay problemas como gasto de bolsillo, la distancia y las horas de apertura incómodas. En algunos casos existen restricciones legales en contra de adolescentes no casados ​​para recibir ciertos servicios y la resistencia de la comunidad para proporcionar información y servicios de SSR a adolescentes, por ejemplo. Los y las adolescentes pueden encontrarse con personal de salud que tienen actitudes críticas y son renuentes a proporcionarles atención relacionada con la SSR. También pueden encontrar que el sistema de salud no asegura la privacidad y la confidencialidad que necesitan. Por el lado de la demanda, los adolescentes pueden no ser conscientes de sus necesidades y pueden tener un sentido de invulnerabilidad que implica no ver la necesidad de buscar servicios de salud.

Los adolescentes en Ecuador enfrentan muchos de los mismos retos y problemas de salud que enfrentan adolescentes de otros lugares. Las causas más comunes de muerte son el suicidio de las adolescentes y las lesiones por accidentes de tráfico en los adolescentes [9]. La violencia, incluida la violencia de pareja, es una causa importante de morbilidad y mortalidad para ambos sexos. El principal problema en términos de salud sexual y reproductiva es la alta tasa de embarazos de adolescentes. En Ecuador, estas altas tasas se extienden a las niñas menores de 15 años de edad [9].

Ecuador ofrece un estudio de caso interesante en salud adolescente porque es un país en transición, y las actitudes y enfoques están cambiando [10]. Los enfoques basados ​​en los derechos han ido ganando terreno y el estado ha reconocido que los y las adolescentes tienen derechos (Art. 35, 39, 44, 45,46 de la Constitución de la República del Ecuador, 2008). Es ampliamente aceptado que los adolescentes necesitan y tienen derecho a servicios e información [11]. Además, Ecuador es un país de ingresos medios que, después de siete años de estabilidad política, está viendo aumentar las inversiones en salud y disminuir la pobreza [9]. El Estado aumentó sus inversiones en salud de US $ 371 millones en 2004 a US $ 1,671 millones en 2012. El gasto actual en salud representa el 8% del Producto Nacional Bruto (PNB), el 52% del gasto público y el resto es privado.

En 2007, se creó el marco del Plan Regional Andino para la Prevención del Embarazo Adolescente [13]. En Ecuador, este plan contribuyó al establecimiento de servicios diferenciados para adolescentes - SADAs (Servicios para atención diferenciada de Adolescentes). Estos servicios deben contar con personal dedicado y capacitado para el cuidado de los adolescentes y un espacio exclusivo y adaptado de acuerdo a la demanda y expectativas de los adolescentes [11]. Sin embargo, en 2011, el Gobierno del Ecuador introdujo una nueva política y decidió implementar un modelo para la prestación de servicios integrados de salud familiar y comunitaria (Modelo de Atención Integral del Sistema Nacional de Salud Familiar e Intercultural (MAIS-FCI)) cuyo principal principio rector era que la atención integrada debía prestarse a lo largo de todo el ciclo de vida. Volveremos al efecto de este cambio en la política en párrafos posteriores.

**Alcance y objetivos del estudio:**

Este artículo pretende describir las necesidades actuales de la atención de la salud de adolescentes en Ecuador, con énfasis en la salud sexual y reproductiva. Se muestra que estas necesidades están siendo atendidas por el actual sistema de salud del Ecuador y se examinan las implicaciones de los cambios recientes en la organización de los servicios de salud para adolescentes, de servicios diferenciados a servicios integrados.

El objetivo es extraer enseñanzas del enfoque ecuatoriano e informar a los encargados de formular políticas, a los directores de programas, a las organizaciones internacionales y a otras partes interesadas sobre la manera de proporcionar una atención de salud amigable para adolescentes – una atención de salud que sea diferenciado e incluya un énfasis en la salud sexual y reproductiva.

**Método**

Los autores buscaron literatura relevante en Pubmed, Popline, y Google Scholar, y en las listas de referencias de documentos identificados como relevantes. Los términos de búsqueda utilizados fueron diferentes combinaciones de las palabras salud, atención de salud, servicios de salud, salud sexual y reproductiva, adolescentes, jóvenes, personas jóvenes y Ecuador. La búsqueda incluyó todas las publicaciones hechas antes del 1 de noviembre de 2014. También fueron consultados - sin límites de tiempo definidos - planes, informes y otra literatura gris publicada por una serie de organizaciones e instituciones, incluyendo el Ministerio de Salud del Ecuador, UNFPA, OPS Y UNICEF. Se incluyeron todas las publicaciones (artículos de revistas y literatura gris que cumplieron con los siguientes criterios: descripciones y/o análisis de comportamientos y problemas de salud de adolescentes en Ecuador, respuesta del sistema de salud a adolescentes en Ecuador, publicado en inglés o español y publicado antes del 1 de noviembre de 2014. Dada la escasez de publicaciones sobre salud de los adolescentes en el país, se utilizaron todas las publicaciones disponibles, excluyendo la literatura que no incluía datos de Ecuador y de adolescentes / jóvenes / personas jóvenes.

Se buscó información complementaria mediante consultas informales con actores clave y el personal de salud en Ecuador en octubre de 2014. En una reunión el primer y segundo autores se reunieron con representantes del UNFPA, UNICEF, OPS, Ministerio de Salud Pública, Ministerio de Desarrollo Social del Ecuador, Organizaciones no gubernamentales (ONG) y dos representantes de jóvenes. Posteriormente se reunieron con el personal de tres centros de salud ecuatorianos - uno en Quito y dos en zonas rurales - y un hospital. Los representantes fueron seleccionados sobre la base de sus conocimientos con la política y el sistema de salud del país y/o familiaridad con la salud de los adolescentes. Los debates se desarrollaron en torno a la situación actual de la salud de adolescentes, los desafíos de adolescentes relacionados con la SSR y la respuesta del sector de la salud a estos temas.

**Resultados**

**Situación de salud de adolescentes en Ecuador y sus necesidades por servicios de salud**

**Accidentes de tráfico, suicidio y violencia**

En 2011, la causa más común de muerte entre las adolescentes fue el suicidio (13% de todas las muertes), en comparación con las lesiones de tráfico entre los adolescentes (19%) [9]. Los accidentes de tráfico fueron la segunda causa de muerte más común entre las adolescentes (8%). Para los varones, la violencia fue la segunda causa más común (12%) y el suicidio la tercera (7%).

**Salud mental y suicidio**

Cada vez hay más reconocimiento en todo el mundo de que la salud mental de los adolescentes ha sido un asunto olvidado [5]. Los indicadores para Ecuador, definitivamente son materia de preocupación. El país es uno de los países con mayores tasas de suicidio entre los jóvenes de la región de las Américas. Además, estas tasas han ido en aumento [14]. Las tasas son más altas para las mujeres que para los hombres en el grupo de 10 a 19 años (7,1% vs 6,4%), pero más altas para los hombres en el grupo de 15 a 24 años (13,0% vs 9,3%). Una encuesta de salud escolar de 2007 entre los estudiantes de 13 a 15 años encontró que el 17% en las ciudades de Guayaquil, el 17,5% en Quito y el 19,4% en Zamora habían considerado intentar suicidarse durante los 12 meses anteriores [15].

**Violencia**Otra preocupación nacional importante es el alto nivel de violencia entre los adolescentes. La encuesta citada anteriormente encontró que 34,8% de los estudiantes en Guayaquil, 36,2% en Quito y 36,1% en Zamora habían sido atacados físicamente una o más veces durante los 12 meses anteriores. Las adolescentes y las mujeres también tienen un alto riesgo de ser expuestas a la violencia por sus parejas. Se estima que la prevalencia de violencia intrafamiliar (IPV) en la vida es del 35% para la violencia física, del 14,5% para la violencia sexual y del 43,4% para la violencia psicológica (INEC 2011, citado en [16]).

**Uso de alcohol**

El consumo de alcohol comienza a menudo temprano entre los adolescentes en Ecuador. En 2008, una encuesta nacional de consumo de drogas entre los estudiantes encontró que la edad media para probar el alcohol por primera vez fue de 12,8 años [17]. En el año 2014, el Ministerio de Salud Pública reportó que el 45,6% de la población ecuatoriana (47,2% de los hombres y 43,9% de las mujeres) entre 10 y 19 años y el 21,3% de los adolescentes entre 10 y 14 años declararon haber probado alcohol. Alrededor del 12,9% de los adolescentes de 10 a 14 años informaron haber estado ebrios al menos una vez. Hasta el 24% de los adolescentes de 13 a 15 años en Guayaquil, 27,7% en Quito y 27,8% en Zamora, dijeron que habían consumido alcohol como para estar "realmente" borrachos una o más veces en su vida [15]. ]. Haber probado alcohol temprano es más común entre los adolescentes en grupos indígenas y aquellos en el quintil más pobre de la población [18].

**Sobrepeso y obesidad**

En 2007, la encuesta realizada en escuelas también encontró que el 28.6% de los estudiantes de 13 a 15 años en Quito tenían sobrepeso y 7.3% eran obesos [15]. Un estudio de un grupo de adolescentes ecuatorianos urbanos y rurales reportó que la prevalencia de dislipidemia, obesidad abdominal y sobrepeso fue de 34,2%, 19,7% y 18,0%, respectivamente, y que el 59% de adolescentes ecuatorianos tienen bajos niveles de aptitud física [19]. Solo un poco más de la cuarta parte de los ecuatorianos entre 10 y 18 años superó el nivel mínimo de actividad física [18].

**Maternidad temprana y salud materna de las adolescentes**

El principal problema nacional relacionado con la SSR de los adolescentes es la alta tasa de embarazos en adolescentes. En 2004, la Encuesta Nacional de Demografía y Salud Materno-Infantil (ENDEMAIN) encontró que más del 20% de las mujeres ecuatorianas encuestadas (de 15 a 49 años) habían tenido un hijo o habían estado embarazadas cuando eran adolescentes [22]. Los embarazos a temprana edad conllevan riesgos elevados de complicaciones tanto para la madre como para el niño, y se consideran un obstáculo para el desarrollo social y económico de las familias y las comunidades [20, 21]. Alrededor del 4% de las defunciones entre las adolescentes del Ecuador se deben a complicaciones durante el embarazo y el parto [9].

De acuerdo con las estadísticas del Instituto Nacional de Estadística y Censos (INEC) de 2010, el 44,1% de las madres tuvieron su primer hijo cuando tenían 15 a 17 años y el 2,4% tuvieron su primer hijo cuando tenían entre 12 Y 14 años. En 2010, un 3,4% de los aproximadamente 3,6 millones de madres en Ecuador tenían entre 12 y 19 años, lo que corresponde a una cifra de 122,301 madres adolescentes [22]. El porcentaje de adolescentes en el grupo de 15 a 19 años de edad con al menos un hijo aumentó de 14,84% en 1990 a 17,53 en 2010 [22]. Los datos sobre los nacimientos de 2013 muestran que el 19,48% de todos los nacimientos corresponden a mujeres de 15 a 19 años, y el 0,76% corresponden a niñas menores de 15 años [22].

**Maternidad temprana, nivel socioeconómico y asistencia escolar**

En 2012, alrededor del 84% de los ecuatorianos entre 15 y 17 años fueron a la escuela, pero tres de cada 10 se quedaron rezagados en sus estudios; es decir, no asistían al grado correspondiente a su edad. De éstos, la mitaderan de comunidades indígenas [22]. La proporción de madres menores de 19 años es más alta en los grupos con bajos ingresos y baja educación. El 47% de los adolescentes de bajo nivel socioeconómico se convirtieron en madres o estuvieron embarazadas durante la adolescencia. Alrededor del 70% de las madres ecuatorianas que quedaron embarazadas durante la adolescencia ya habían abandonado la escuela antes de quedar embarazadas. Del 30% restante, alrededor del 12% continuó sus estudios, mientras que el 18% interrumpió sus estudios debido al embarazo. Al parecer, el 70% de los que interrumpieron sus estudios no regresó a la escuela [22].

**Actividad sexual y uso de anticonceptivos**

Como es de esperar, el alto número de adolescentes embarazadas se debe a una combinación de actividad sexual temprana y uso bajo o inadecuado de la anticoncepción. La encuesta ENSANUT en 2012 encontró que casi seis de 10 mujeres entre 15 y 24 años habían tenido una experiencia sexual. Un 54,6% de ellos había tenido esta experiencia antes de casarse. Esto representa un aumento considerable con respecto a 2004, cuando sólo el 46,7% de los jóvenes de entre 15 y 24 años habían tenido una experiencia sexual y el 37,2% informó haber tenido una experiencia sexual antes del matrimonio [22]. La encuesta en la escuela citada anteriormente encontró que 26,0% de los niños y 7,1% de las niñas de 13 a 15 años de edad en Guayaquil habían tenido relaciones sexuales al menos una vez [15]. Las cifras correspondientes fueron 23,4% de los niños y 8,1% de las niñas en Quito y 33,7% de los niños y 9,9% de las niñas en Zamora habían tenido relaciones sexuales al menos una vez.

Según el estudio ENDEMAIN de 2004, casi todos los adolescentes (97%) conocían los métodos modernos de anticoncepción; el 47% las utilizaba en el momento de la encuesta y el 13,5% las utilizó durante su primera relación sexual (22). En el momento de la encuesta ENSANUT 2012, el porcentaje de adolescentes (15 a 19 años) que reportaron usar anticonceptivos había aumentado a 68,9% [23]. En general, el uso de anticonceptivos modernos entre los adolescentes casi se ha triplicado en las últimas dos décadas [23]. Un estudio nacional encontró que el 89% de los que eran sexualmente activos antes de los 17 años no habían usado condón durante su primer encuentro sexual (González-Rozada, 2010 citado en MCDS 2014).

Un estudio realizado en seis escuelas secundarias de la ciudad de Cuenca, Ecuador, y en 20 escuelas secundarias de Cochabamba, Bolivia, encontró que adolescentes sexualmente activos (entre 14 y 18 años) que consideran la igualdad de género como importante reportaron un mayor uso actual de anticonceptivos con las parejas que habían formado. También eran más propensos a describir su última relación sexual como una experiencia positiva y consideraron más fácil hablar con sus parejas acerca de la sexualidad, en comparación con los adolescentes sexualmente experimentados que estaban menos inclinados a temas de igualdad de género.

**Violencia sexual**

Las investigaciones demuestran que un número relativamente elevado de relaciones sexuales resulta de la violencia o son aceptadas en circunstancias en las que alguno de la pareja, normalmente la adolescente, siente que no puede negarse [25]. Un 14,6% de las mujeres ecuatorianas de 15 a 49 años informaron en el 2004 que habían sufrido violencia sexual en sus vidas [25]. Menos de un tercio (27.6%) de los estudiantes de una encuesta de la escuela secundaria realizada en Rumiñahui en la provincia de Pichincha creían que era aceptable rechazar el sexo en cualquier momento y menos de un cuarto (23.4%) de los estudiantes creía que no era aceptable rechazar el sexo bajo ninguna circunstancia, mientras que la mitad (49,0%) creía que el derecho a negarse dependía de la situación [26].

**Asociación entre violencia de género y embarazo temprano**

Parece haber una asociación entre la violencia basada en el género y el embarazo antes de los 18 años. La violencia entre las parejas íntimas es más común entre las que quedan embarazadas en temprano en la adolescencia que entre las que lo hacen más tarde. Según un informe gubernamental publicado en 2014, las mujeres que quedaron embarazadas durante la adolescencia tienen entre 1.55 y 1.66 veces más probabilidad de ser abusadas física o psicológicamente por sus parejas o ex compañeros que las mujeres que no experimentaron embarazos durante la adolescencia [22]. Haber sufrido de abuso sexual durante la infancia o la adolescencia fue un factor de riesgo para el embarazo adolescente según un estudio realizado en la cuenca del Amazonas [27]. Del mismo modo, un estudio hospitalario en Quito encontró que el abuso sexual era más de tres veces más común entre las adolescentes embarazadas en comparación con las adolescentes que no estaban embarazadas (14,9% vs 4,5%) [28].

**Los grupos indígenas son más vulnerables**

Un 9% de los ecuatorianos de 0 a 17 años pertenecen a grupos indígenas y el 8% pertenecen a grupos afro-ecuatorianos [9]. Los adolescentes de estos grupos tienden a tener unapeores indicadores de salud, educación, acceso a servicios y pobreza. Por ejemplo, los miembros de estos grupos tienen tasas más altas de abandono escolar, embarazo en la adolescencia, infecciones de transmisión sexual (ITS), incluido el VIH; menores tasas de atención médica calificada durante el parto y mayores tasas de consumo de tabaco y alcohol [ 29]. Alrededor del 66,7% de las adolescentes de 15 a 19 años de la provincia amazónica de Orellana estaban embarazadas en la encuesta de 2004, en comparación con el 13,9% de adolescentes de la provincia más poblada de Guayas (ENDEMAIN 2004, citado en Cabrero et al. 2010).

**Cuestiones de género**

La desigualdad de género, tanto para los adolescentes como para las adolescentes, se ve intensificada por las normas culturales (el "machismo") que espera que los adolescentes sean agresivos y dominantes y que las adolescentes sean sumisas y obedientes. Esto limita su capacidad para protegerse y los/las deja vulnerables a una amplia gama de riesgos para la salud.

Lo que podemos concluir de los datos presentados anteriormente es que los adolescentes en Ecuador tienen un alto riesgo de estar expuestos a la violencia, tienen niveles preocupantes de enfermedad mental y son vulnerables al abuso de alcohol, la obesidad y la actividad física insuficiente. Además, hay un patrón de debut sexual temprano con una falta o conocimiento limitado sobre cómo protegerse y prevenir embarazos no deseados, llevando al aumento de la fecundidad adolescente. Investigaciones internacionales han demostrado que estos temas a menudo están conectados. Por ejemplo, el consumo de alcohol y la violencia de la pareja íntima tienden a asociarse con el debut sexual temprano y el bajo uso de la anticoncepción [30]. La investigación sobre el suicidio femenino sugiere que la violencia de pareja y el embarazo adolescente son factores de riesgo y que las mujeres que se suicidan son más propensas a haber sufrido abuso sexual [14]. La situación de los adolescentes en Ecuador destaca la necesidad de llegar a ellos con información de salud y lograr que los servicios de salud sean amigables y, por lo tanto, atractivos para este grupo de edad.

**Uso de servicios de salud por adolescentes en Ecuador**

Desde 2007, el Gobierno del Ecuador ha trabajado para que el acceso a la atención de la salud sea gratuito y ha habido un aumento considerable en el uso de los servicios de salud. Los datos relativos específicamente al uso de los servicios de salud por parte de los adolescentes son algo limitados. Sin embargo, existen estadísticas sobre el número de adolescentes de 10 a 14 años de edad y de 15 a 19 años que acudieron a los centros de salud para recibir servicios de prevención y curación. Como puede verse en las figuras 1 y 2, los números han aumentado constantemente.

Figura 1: Consultas de morbilidad (primera y subsiguiente)

[Inserte la figura 1]

Figura 2: Consultas preventivas (primera y subsiguiente).

[Inserte la figura 2]

Para más información específica sobre servicios, la asistencia calificada del parto es uno de los pocos indicadores de los que se dispone. En los últimos años, las mujeres jóvenes han sido más frecuentemente atendidas por personal calificado durante el parto. Como se señaló anteriormente, el embarazo, el parto y las condiciones posparto son, las razones más comunes para la hospitalización de adolescentes (de 10 a 17 años); alrededor del 50% de las hospitalizaciones de adolescentes están relacionadas con estos eventos [9].

**Fuentes de información**

Los adolescentes tienen un acceso limitado a la información y la información en materia de salud entregada por adultos. Una encuesta realizada en siete escuelas secundarias en 2005 en la provincia de Pichincha encontró que la fuente más importante de información general sobre la SSR fueron los medios de comunicación (83% de los encuestados). Sólo el 14% informó que recibió información de la escuela. El 60,9% respondió que había conversado con sus padres sobre SSR, pero sólo el 11% citó a sus padres como fuentes de información. La falta de confianza y vergüenza se dieron como las principales razones para no discutir sobre SSR con los padres. El dos por ciento reportó obtener información de SSR de sus amigos. Cuando se trataba de información, más específicamente de planificación familiar, la escuela era la principal fuente de información (23%) [26]. Mientras que sólo una pequeña proporción de adolescentes informó haber recibido información sobre la SSR de sus padres. Una encuesta nacional del UNFPA sobre las percepciones acerca de la planificación familiar y la anticoncepción encontró que la gran mayoría de la población adulta está a favor de dar a los adolescentes acceso a la anticoncepción y a favor de proveer información sobre su uso a adolescentes [31].

**Las barreras (estructural, económica, sociocultural) al uso de los servicios de SSR y el acceso a la información**

Adolescentes y jóvenes se enfrentan a una amplia gama de barreras que pueden limitar su acceso a los servicios de salud. Un estudio realizado en nombre del Comité Regional Andino para la Prevención del Embarazo Adolescente, dividió las barreras de acceso en tres grupos principales: sociocultural, institucional y político (nacional o territorial) [7]. El último grupo incluye barreras legales y regulatorias. La escasa disponibilidad de servicios de salud, los altos costos, la baja calidad y la falta de recursos -incluidos los recursos humanos- son algunas de las principales barreras del lado de la oferta.

La adolescencia es comúnmente vista en Ecuador como un período propenso a riesgos y problemas, especialmente en materia de sexualidad. A pesar de que los datos del UNFPA presentados anteriormente indican que muchas personas aceptan que los adolescentes deben poder acceder a la anticoncepción, los adolescentes suelen encontrar normas estrictas y actitudes moralistas de los adultos [9]. Las relaciones de género se caracterizan por el machismo (sexismo), la homofobia y la expectativa de que las mujeres lleguen a ser madres temprano en sus vidas. En lugar de proporcionar información y alentar/apoyar a sus hijas para que se protejan de los problemas de salud sexual y reproductiva, los padres y tutores tienden a tratar de evitar que las mujeres jóvenes tengan contacto con hombres [9]. Para muchos ecuatorianos, la única manera aceptable para que las mujeres jóvenes eviten el embarazo es que "no vayan con hombres". El estudio nacional de 2011 encontró que el 68% de las mujeres y el 61% de los hombres no aprobaban las relaciones sexuales entre los adolescentes [31]. Estas actitudes también son comunes entre los trabajadores de la salud [7, 32, 33]. Ante esto, una de las principales barreras para el acceso de adolescentes a la anticoncepción -y una de las principales razones de la brecha entre el conocimiento sobre la anticoncepción y su uso real- es la actitud de los trabajadores de salud que creen que la anticoncepción no es una respuesta adecuada a la actividad sexual en adolescentes [22].

Al mismo tiempo, la maternidad es el horizonte natural para muchas mujeres jóvenes, y en particular para las de las regiones más pobres, ya que la maternidad les permite ser reconocidos material y simbólicamente por sus familias, comunidades y el Estado. La maternidad también otorga a las mujeres jóvenes derechos y servicios garantizados por el Gobierno [22]. Los estudios han encontrado que muchas adolescentes creen que para ellas no hay alternativas a la maternidad temprana que les permita ser reconocidas como adultos y personas responsables [7, 34]. Esta confluencia entre el acceso limitado a los servicios y la información sobre salud sexual y reproductiva, por un lado, y la idealización de la maternidad, por otro, deja a los y las adolescentes vulnerables no sólo a los embarazos tempranos, sino también a las infecciones de transmisión sexual y a la violencia sexual.

**Introducción y ampliación de la atención sanitaria diferenciada para los jóvenes (2007-2011)**

La siguiente sección describe el desarrollo en Ecuador de servicios de salud diferenciados para adolescentes. Este enfoque diferenciado fue concebido para responder a los desafíos y necesidades descritos anteriormente. En 1988, Ecuador inició un proceso histórico con la apertura de un Servicio de Atención Integral para adolescentes embarazadas en el Hospital Gíneco-Obstétrico Isidro Ayora de Quito. El establecimiento de este servicio visibilizó la demanda y la necesidad de servicios de salud entre adolescentes. En 1992, el Programa Nacional de Salud de los Adolescentes elaboró un manual de normas y procedimientos para la atención integral de la salud de adolescentes. El manual fue desarrollado por profesionales médicos y empleó un enfoque biomédico [10]. En 2005, la Política Nacional de Salud Sexual y Salud Reproductiva identificó los embarazos de adolescentes como un tema prioritario y afirmó que los servicios para adolescentes debieran fortalecerse [35]. Sin embargo, hasta 2007, sólo tres unidades de salud pública, distintas del hospital Isidro Ayora, proporcionaban atención integral diferenciada para adolescentes [7]. Los servicios amigables para atención integral diferenciada de adolescentes del Hospital Gíneco-Obstétrico Isidro Ayora en Quito, así como tres unidades de nivel primario, se iniciaron y continuaron por iniciativa de personas y grupos particulares interesados ​​en abordar los problemas de salud de adolescentes. El modelo no se expandió ni se replicó hasta 2007 debido principalmente a la falta de voluntad política.

En 2007 se lanzó el Plan Andino y el Plan Nacional de Prevención del Embarazo de Adolescentes [13] y en 2009, las nuevas Guías para el Cuidado Integral de la Salud de Adolescentes [11]. El Plan Nacional de Prevención del Embarazo en Adolescentes utiliza un enfoque basado en los derechos y se basa en la suposición de que para que los y las adolescentes puedan ejercer sus derechos reproductivos, no solo necesitan tener acceso a una red de servicios, sino que deben tener el poder para tomar control de su sexualidad. Una de las principales cuestiones abordadas en el plan era la necesidad urgente de información a los grupos vulnerables o marginados que requieren atención prioritaria. Estos incluyeron adolescentes menores (10-14 años); adolescentes con poca educación; adolescentes que no asisten a la escuela; rurales e indígenas; los que viven en zonas aisladas del país; así como a adolescentes migrantes, desplazados internos y refugiados.

Las Normas y Procedimientos para la Atención Integral de Salud a Adolescentes del 2009 promovieron la atención basada en: (i) servicios diferenciados prestados con enfoque integral, intercultural, participativo y enfoque de derechos; y (ii) una atención amigable caracterizada por el respeto, la confidencialidad, las actitudes positivas de los trabajadores de la salud y las aptitudes y competencias apropiadas entre dichos trabajadores. El modelo de atención de salud diferenciada se denominó Servicios de Atención Diferenciada para Adolescentes (SADA). Este nuevo enfoque tenía como objetivo facilitar el acceso de los adolescentes (10-19 años) a los servicios de salud. El Ministerio de Salud Pública promovió la implementación de servicios diferenciados para la atención integral de adolescentes en unidades de salud de primer nivel y en hospitales, a partir de un "paquete normativo" para tales cuidados (normas, protocolos, y estándares de calidad) y en la sensibilización y capacitación del personal multidisciplinario de los establecimientos de salud. Las directrices mencionadas anteriormente establecen que los servicios diferenciados deben incluir "personal capacitado a tiempo completo que preste atención basada en un enfoque de derechos, sensible al género, culturalmente sensible, participativo e integrado", y que debe hacerlo en un "lugar adaptado a las necesidades y preferencias de los adolescentes, y asignado a su uso exclusivo"[11].

Para abordar el problema de los embarazos precoces, el Gobierno formuló en 2011 la Estrategia Nacional Intersectorial de Planificación Familiar y Prevención de Embarazo Adolescente (ENIPLA) para actuar en cuatro áreas: 1) mantener a los adolescentes en el sistema educativo y fortalecer la educación sexual integral; 2) mejorar el acceso de los adolescentes a los servicios de SSR incluyendo métodos anticonceptivos; 3) acción familiar y comunitaria, diálogo social y corresponsabilidad; y 4) Promover cambios en los patrones socioculturales.

También en 2011, se anunció un cambio importante en la política ya que el Ministerio de Salud Pública lanzó un modelo para la prestación de servicios integrados de salud familiar y comunitaria (Modelo de atención integral del sistema nacional de salud familiar comunitario e intercultural (MAIS-FCI). MAIS conceptualiza la atención de calidad bio-psico-social con un énfasis en la prevención y promoción que presta atención a cada persona de manera integrada y apoya el desarrollo humano. El marco de MAIS da prioridad a la atención integrada durante todo el ciclo de vida y a la medicina familiar y comunitaria, lo que en consecuencia, significó la abolición de los programas verticales. Como era de esperar, esto tuvo un efecto en el enfoque diferenciado adoptado para los adolescentes. A nivel nacional, el programa de adolescentes fue desautorizado y, a nivel local, el proceso de transición se vio afectado por la falta de orientación del nuevo modelo en cuanto a la gestión de los SADAs. Sin embargo, el Plan Nacional de Buen Vivir (2013-2017) hace un llamado a garantizar el acceso efectivo a servicios integrados de salud sexual y reproductiva [9].

**Logros del programa de servicios diferenciados**

El Ministerio de Salud Pública, en colaboración con el UNFPA, realizó una encuesta sobre el estado y los logros de los SADAs en 2012 [36]. Se recibieron respuestas de 74 de los 158 SADAs, cuyos informes sugirieron que estaban en vigor en 2011 (tasa de respuesta del 46,8%) y de 14 de 24 provincias (una tasa de respuesta del 58%). Además, se recibieron respuestas de 26 nuevos centros de salud que habían iniciado SADAs durante el año anterior a la encuesta, con lo que el número total de SADAs que respondieron fue de 100 (de un total de 184). Algunos de los SADAs que no respondieron supuestamente dejaron de ofrecer servicios diferenciados para adolescentes debido a escasez de personal o su reorganización.
Los resultados de la encuesta mostraron que el número de SADAs aumentó gradualmente de 38 en 2008 a 97 en 2009, a 139 en 2010, a 158 en 2011. En 2011, las 24 provincias tuvieron atención diferenciada para adolescentes. Había solamente dos a tres SADAs en cada una de cinco provincias y cuatro SADAs en cada una de otras nueve provincias. Además, había 27 SADAs en Guayas, 29 en Pichincha y 15 en Manabí. Esto significa que estas tres provincias, las más pobladas del Ecuador, en conjunto tenían el 52,5% de la población del país en 2010 y contaban con el 38,5% de los SADAs en 2012 [36].

De las 14 provincias que respondieron, 11 reportaron aumentos significativos en el uso de servicios por parte de adolescentes, y dos informaron una disminución (Pichincha, un 30% menos y Cotopaxi, un 9%). De 2008 a 2011, el número de servicios de prevención ofrecidos aumentó en estas 100 unidades, de 138.787 visitas a 264.924, lo que representó un aumento del 90,9%. Sin embargo, una gran parte (57,5%) de este aumento se produjo en una provincia: Guayas, que tiene el 25% de la población del país, reportando 72.521 consultas más en 2011 (de 63.792 visitas a 136.313). Esto significa que para el resto del país, el aumento fue del 71,5% (53.616 consultas más, de 74.995 a 128.611). La tendencia fue la misma para los otros dos tipos de servicios cubiertos por la encuesta (consultas de morbilidad y embarazo) [36].
La encuesta encontró que en 2011, 1.061 profesionales de la salud fueron sensibilizados y capacitados para atender a adolescentes en estas 100 unidades. Un tercio (32%) eran médicos, 20% eran obstetras, 14% eran enfermeras u odontólogos, 4% eran psicólogos, y, 2% eran trabajadores sociales o nutricionistas [36].

En cuanto a la sensibilización de la comunidad sobre los SADAs y la necesidad de prevenir los embarazos de adolescentes, se informó que 63.004 personas habían asistido a actividades educativas (casi un tercio en Guayas). Estas actividades fueron ofrecidas a adolescentes (en el 83% de los casos), a padres y madres (11%), a maestros (2%) y a otros como policías, bomberos, funcionarios de la defensa civil y líderes comunitarios 4 %) [36]. La encuesta no recopiló datos para conocer en qué medida se beneficiaron los grupos desfavorecidos.
Los elementos y logros descritos preceden a la institucionalización de la atención integrada a los adolescentes en el primer nivel de MAIS-FCI. La justificación de este nuevo enfoque, como se señaló anteriormente, era reducir las tendencias a la fragmentación y segmentación de servicios y sistemas que comprometen la calidad de la prestación de servicios y generan desigualdades en el acceso a los servicios. Esto significó un paso de la atención diferenciada para grupos particulares hacia un enfoque en el que todos los grupos -en teoría - recibirían el mismo nivel de atención a lo largo de su ciclo de vida.

En consecuencia, se suspendió el desarrollo de capacidades, los sistemas de apoyo y el seguimiento de los SADAs. También significa que las autoridades sanitarias locales en muchos casos dejan de considera a los adolescentes como un grupo que debe recibir atención diferenciada. Esto condujo rápidamente a una situación en la que el personal que había sido entrenado para cuidar a los adolescentes, se sentía ignorado y sin rumbo, y ya no se encontró en una posición donde pudieran usar las habilidades para las cuales habían sido entrenados. Las entrevistas con personal de salud que había recibido formación para ofrecer servicios diferenciados a los adolescentes indicaron que posteriormente tenían que trabajar con todos los grupos de pacientes y estaban confundidos acerca de sus funciones [36].
Las consultas mantenidas con el personal de salud en el contexto de este estudio confirmaron que algunos de los problemas causados ​​por la transición de SADA a MAIS fueron: incertidumbre relacionada con las competencias necesarias para atender a la población en general; falta de habilidades técnicas, discontinuidad a nivel central de la gestión, el seguimiento de la formación y la implementación, pérdida de datos e indicadores recogidos por los SADAs, y el desmantelamiento de los SADAs por parte de autoridades locales. Según se informa, las instalaciones en algunos lugares siguen ofreciendo atención diferenciada a los adolescentes dentro del marco del MAIS, al mismo tiempo que amplían los servicios a la población en su conjunto. Sin embargo, a veces esta atención diferenciada continua se lleva a cabo en condiciones difíciles, debido a la pérdida de disponibilidad de tiempo y espacios de trabajo dedicados a adolescentes. La continuidad de la atención dirigida a los adolescentes depende en gran medida de la presencia de personas comprometidas y de la gestión local que esté dispuesta a asignar prioridad a los jóvenes.

Mientras tanto, los datos sobre la aceptación de los servicios y sobre cómo ha sido percibida la transición del modelo anterior por los adolescentes son escasos. Una razón importante para ello es que los informes ya no se recogen del sistema de información establecido previamente por los SADAs.

**Discusión**Los hallazgos de este estudio ponen de relieve la necesidad urgente de una atención de la salud diferenciada y respetuosa con los jóvenes en el Ecuador. Los y las adolescentes son vulnerables a una variedad de problemas de salud, incluyendo embarazo temprano y no deseado, violencia de pareja, abuso de alcohol y drogas, depresión y suicidio. Sin embargo, tienen un acceso limitado a servicios preventivos, curativos y de rehabilitación adecuados. Algunas de las barreras más importantes son socioculturales y actitudinales, tanto entre la población en general como entre los trabajadores de la salud. Estas barreras han llevado a la pobre demanda de adolescentes a servicios de SSR.

El caso del modelo SADAs en Ecuador ofrece algunas lecciones interesantes. En primer lugar, demostró que existía una demanda insatisfecha de servicios diferenciados para los jóvenes. En segundo lugar, demostró que era posible satisfacer esta demanda ofreciendo atención integral diferenciada que responda a las necesidades específicas de los adolescentes. El enfoque fue eficaz, al menos en términos de aumentar la aceptación de los servicios de salud, en parte porque garantizaban la confidencialidad, eran amigables y respetuosos, y crearon confianza entre las personas de este grupo de edad. A lo largo de tres años, el número de SADAs aumentó de 38 a 158. Se logró la cobertura nacional - al final, hubo SADAs en las 24 provincias de Ecuador, seguido de un considerable aumento en el número de adolescentes que acudieron a servicios preventivos y cuando estaban heridos o enfermos. Estos jóvenes también acudieron a los servicios de atención prenatal y de parto. Los 100 SADAs que respondieron a la encuesta de 2012 reportaron casi duplicar el número de adolescentes que buscaban servicios preventivos entre 2008 y 2011 [36].

El cambio posterior en Ecuador de un modelo de salud dirigido a diversos períodos del ciclo de vida con programas específicos a un modelo de atención integral demostró la fragilidad de los logros. Cuando se modificó la estructura institucional y el sistema de apoyo a los adolescentes, el personal capacitado para ofrecer atención diferenciada ya no era capaz, en muchos casos, de prestar atención a los adolescentes. Goicolea et. al. quienes estudiaron tres SADAs en 2010, preveían esta situación cuando observaban que la sostenibilidad de los servicios amigables para adolescentes y jóvenes se veía amenazada por una falta de financiación estable y por falta de estructuras habilitadoras claras en términos de formación y apoyo [10].

Además, a raíz de los cambios, los establecimientos de salud ya no estaban obligados a informar sobre las actividades y servicios específicos de adolescentes, y el Ministerio de Salud Pública no recopila indicadores que permitan evaluar el alcance del uso posterior de los servicios de salud por parte de adolescentes o si están satisfechos con los servicios ofrecidos bajo el actual modelo MAIS. Como también observó Goicolea et. al. (2012), los autores del presente informe señalan una reciente falta de datos precisos, combinados con rutinas inconsistentes para registrar datos. La falta de monitoreo y evaluación de los servicios diferenciados que se ofrecen a los jóvenes en Ecuador refleja la situación en muchos países [6, 8]. En consecuencia, dentro del sistema actual, las necesidades específicas de los adolescentes son, en gran medida, no visibles y no reconocidas. Esto conlleva al riesgo que las respuestas a sus necesidades y problemas no sean adecuadas.

**Conclusión**

Este estudio confirma claramente lo observado por otros: los adolescentes necesitan tener acceso a servicios integrales diferenciados de calidad, amigables con los adolescentes, proporcionados por médicos y personal capacitados para trabajar con este grupo de población. Los proveedores de servicios deben ser capacitados para que comprendan las demandas y necesidades de adolescentes y jóvenes, sepan cómo comunicarse con ellos y los asesoren eficazmente, y sepan prestar servicios de manera no amenazadora [37]. La formación continua y el apoyo son esenciales para que los proveedores de salud se sientan lo suficientemente seguros como para implementar un servicio que puede no ser familiar para ellos [10]. Las estrategias para los servicios amigables con los adolescentes deben adaptarse a las necesidades de desarrollo de este grupo de edad y al contexto social, y deben emplear enfoques multifacéticos. Es esencial continuar la inversión en estrategias eficaces de prevención y tratamiento [4]. Como ha señalado la OMS, para que los servicios de salud sean adecuados para los jóvenes, deben estar disponibles, accesibles, aceptables y equitativamente ofrecidos a diferentes subpoblaciones juveniles [38].

El estudio también destaca la necesidad de mecanismos de rendición de cuentas, sistemas de monitoreo rigurosos e indicadores relacionados con el acceso de los adolescentes a los servicios de salud. Esta información debe extenderse más allá de las cifras sobre la asistencia calificada en el parto y las necesidades insatisfechas de anticoncepción.

A pesar del aumento de la inversión y de la mejora general de los servicios de salud en el Ecuador, existe una necesidad urgente de intensificar los esfuerzos para proporcionar atención eficaz a los adolescentes. Las barreras que actualmente limitan la provisión de tal cuidado y restringen su uso deben ser eliminadas. Muchos adolescentes no se sienten bienvenidos o cómodos en la búsqueda de servicios de salud que - en su percepción - no siempre garantizan la confidencialidad. Entre otras cosas, las barreras afectan el acceso y el uso de los servicios de SSR y la anticoncepción. El mejor enfoque parece ser tomar medidas para garantizar atención diferenciada e integral a los adolescentes. Dichos servicios de salud deben reflejar las necesidades de confidencialidad y privacidad de los adolescentes, y deben ofrecerse de una manera amigable e inclusiva. En el marco de la estrategia de promoción de la planificación familiar, el Ministerio de Salud Pública ha procurado mejorar el acceso a la anticoncepción gratuita y proporcionar al personal sanitario las herramientas y habilidades necesarias para que puedan proporcionar asesoramiento sobre anticonceptivos. Sin embargo, es un desafío para Ecuador responder de manera integrada a los problemas del embarazo temprano, la salud mental y la violencia, los cuales ocurren en altas tasas entre los adolescentes. Otra tarea desafiante es mantener una atención diferenciada en un entorno de reestructuración hacia un modelo de servicios para todos los segmentos de la población. A pesar de las buenas intenciones, el modelo recientemente adoptado condujo a la interrupción de importantes elementos del programa preexistente para la atención diferenciada de la salud de los adolescentes, incluidas las funciones de apoyo a la creación de capacidad y de apoyo centralizadas.

Ecuador es un país en transición demográfica. Para mejorar las oportunidades de los adolescentes y los jóvenes, se debe desarrollar e implementar una política que considere sus necesidades específicas de salud, educación y desarrollo económico. Una política de este tipo debe llamar tanto a los esfuerzos intersectoriales como a la participación de los jóvenes. En los procesos de transición del sistema de salud, como el que ha vivido el Ecuador, deben salvaguardarse estrategias para superar los obstáculos a la provisión y uso de servicios de salud por parte de adolescentes. Es importante mantener las competencias y la moral del personal capacitado para la atención de adolescentes a través de la capacitación y el apoyo continuo e informar a estos trabajadores de la salud sobre los cambios en el sistema para que puedan seguir ofreciendo atención de calidad. También son esenciales las normas y estándares que permiten el monitoreo y la retroalimentación, así como el mantenimiento de los canales de comunicación y el mantenimiento de los sistemas de información para identificar tanto las buenas prácticas como las dificultades encontradas en la atención integral a los adolescentes.
Por último, es necesario introducir en los planes de estudios de los cursos de pre y posgrado de las disciplinas pertinentes (medicina, obstetricia, psicología, educación, trabajo social y derecho), cuestiones relacionadas con los adolescentes y sus derechos, en particular sus derechos en materia de sexual y salud reproductiva. La educación integral en sexualidad también debe incluirse en estos currículos con el objetivo de asegurar que dichos profesionales se gradúen con los conocimientos, la comprensión y las habilidades necesarias para proporcionar servicios eficaces a adolescentes.

**Abreviaturas**ENDEMAIN: Encuesta Nacional de Demografía y Salud Materna e Infantil

ENIPLA: Estrategia Nacional Intersectorial de Planificación Familiar y Prevención de Embarazo en Adolescentes

VIH: Virus de Inmunodeficiencia Humana

INEC: Instituto Nacional de Estadística y Censos

MAIS-FCI: Modelo de atención integral del sistema nacional de salud familiar comunitaria e intercultural

MCDS: Ministerio Coordinador de Desarrollo Social

MSP: Ministerio de Salud Pública

ONG: Organización No Gubernamental

SADA: Servicios para Atención Integral Diferenciada de Adolescentes

SSR: Salud Sexual y Reproductiva

ITS: Infección de transmisión sexual

UNFPA: Fondo de Población de las Naciones Unidas

UNICEF: Fondo de las Naciones Unidas para la Infancia

OMS: Organización Mundial de la Salud

**Aprobación de ética y consentimiento para participar**

No aplica

**Consentimiento para la publicación**

No aplica

**Disponibilidad de datos y material**

No aplica

**Conflicto de intereses**

Los autores declaran que no tienen conflicto de intereses.

**Fondos**El estudio fue apoyado por la OMS y Family Care International.

**Contribuciones de autor**

Venkatraman Chandra-Mouli concibió la idea de esta documentación y participó en su diseño y revisión. Joar Svanemyr dirigió la revisión sistemática y la redacción del manuscrito. Susana Guijarro, Betzabe Butron Riveros contribuyeron en la recopilación de datos, redacción, revisión y edición del manuscrito. Todos los autores leyeron y aprobaron el manuscrito final. Lucia Gómez Garbero ayudó a revisar y hacer las revisiones apropiadas al borrador final del manuscrito.

**Reconocimientos**

Queremos agradecer al Ministerio de Salud Pública de Ecuador por haber acordado trabajar con nosotros y proporcionar toda la información necesaria. Este estudio cuenta con el apoyo del Departamento de Salud Reproductiva e Investigación de la Organización Mundial de la Salud, UNFPA y Family Care International.

**Referencias**

1. Health for the World’s Adolescentshttp://apps.who.int/adolescent/second-decade/. Accessed 1 November 2014

2. Patton GC, Coffey C, Cappa C, Currie D, Riley L, Gore F, Degenhardt L, Richardson D, Astone N, Sangowawa AO, Mokdad A, Ferguson J: Health of the world’s adolescents: a synthesis of internationally comparable data. Lancet 2012;379:1665–1675.

3. Bearinger LH, Sieving RE, Ferguson J, Sharma V: Global perspectives on the sexual and reproductive health of adolescents: patterns, prevention, and potential. Lancet 2007;369:1220–1231.

4. Chandra-Mouli V, Svanemyr J, Amin A, Fogstad H, Say L, Girard F, Temmerman M: Twenty Years After International Conference on Population and Development: Where Are We With Adolescent Sexual and Reproductive Health and Rights?J Adolesc Heal. 2015;56:1–6.

5. Kieling C, Baker-Henningham H, Belfer M, Conti G, Ertem I, Omigbodun O, Rohde LA, Srinath S, Ulkuer N, Rahman A: Child and adolescent mental health worldwide: Evidence for action. Lancet 2011;378:1515–1525.

6. Denno DM, Hoopes AJ, Chandra-Mouli V: Effective Strategies to Provide Adolescent Sexual and Reproductive Health Services and to Increase Demand and Community Support. J Adolesc Heal 2015;56:S22–S41.

7. Mejía ML, Montoya P, Blanco AJ, Mesa ML, Moreno D, Pacheco CI: Barreras Para El Acceso de Adolescentes Y Jóvenes a Servicios de Salud. Propuesta Para Su Identificación Y Superación. UNFPA; 2010.

8. Tylee A, Haller DM, Graham T, Churchill R, Sanci LA: Youth-friendly primary-care services: how are we doing and what more needs to be done?Lancet 2007;369:1565–1573.

9. CNII: La Niñez Y Adolescencia En El Ecuador Contemporáneo: Avances Y Brechas En El Ejercicio de Derechos. Quito: Consejo Nacional para la Igualdad Intergeneracional; 2014.

10. Goicolea I, Coe A, Hurtig A, San Sebastian M: Mechanisms for achieving adolescent-friendly services in Ecuador: a realist evaluation approach.Glob Health Action 2012;5:1–14.

11. MSP: Normas Y Procedimientos Para La Atención Integral de Salud a Adolescentes [Guidelines for Integral Care for Adolescents]. Quito: Ministerio de Salud Público; 2009.

12. Global health expenditure databasehttp://apps.who.int/nha/database/ViewData/Indicators/en. Accessed 15 November 2014.

13. MSP: Plan Andino de Prevención de Embarazo En Adolescentes [Andean Plan for Adolescent Pregnancy Prevention]. Quito: Ministerio de Salud Público; 2007.

14. Quinlan-Davidson M, Sanhueza A, Espinosa I, Escamilla-Cejudo JA, Maddaleno M: Suicide among young people in the Americas. J Adolesc Heal. 2014;54:262–268.

15. MSP: Global School-Based Student Health Survey. 2007 Ecuador Factsheet. Quito: Ministerio de Salud Público; 2007.

16. Hidrobo M, Peterman A, Heise L: The Effect of Cash, Vouchers and Food Transfers on Intimate Partner Violence: Evidence from a Randomized Experiment in Northern Ecuador. Washington DC: International Food Policy Research Institute; 2013.

17. CONSEP: Encuesta Nacional Sobre Consumo de Drogas En Estudiantes de Ensenanza Media. Quito: Consejo Nacional de Control de Sustancias Estupefacientes; 2008.

18. Freire W, Belmont P, Gómez L, Mendieta M, Monge R, Piñeiros P, Ramírez-Luzuriaga M, Romero N, Sáenz K, Silva-Jaramillo K: Encuesta Nacional de Salud Y Nutrición. ENSANUT-ECU 2012. Tomo I. Quito: Ministerio de Salud Pública del Ecuador and Instituto Nacional de Estadística y Censos; 2014.

19. Andrade S, Ochoa-Avilés A, Lachat C, Escobar P, Verstraeten R, Van Camp J, Donoso S, Rojas R, Cardon G, Kolsteren P: Physical fitness among urban and rural Ecuadorian adolescents and its association with blood lipids: a cross sectional study.BMC Pediatr 2014;14:106.

20. UNFPA: Adolescent Pregnancy: A Review of the Evidence. New York: UNFPA; 2013.

21. Ganchimeg T, Ota E, Morisaki N, Laopaiboon M, Lumbiganon P, Zhang J, Yamdamsuren B, Temmerman M, Say L, Tunçalp Ö, Vogel J, Souza J, Mori R: Pregnancy and childbirth outcomes among adolescent mothers: a World Health Organization multicountry study. BJOG An Int J Obstet Gynaecol 2014;121:40–48.

22. MCDS: Investigación Cuantitativa Y Cualitativa: ¿Por Qué Aumenta El Embarazo En Adolescentes? Quito: Ministerio Coordinador de Desarrollo Social; 2014.

23. INEC/MSP: Encuesta Nacional de Salud Y Nutrición ENSANUT 2012. Quito: Instituto Nacional de Estadísticas y Censos/Ministerio de Salud Pública; 2012.

24. De Meyer S, Jaruseviciene L, Zaborskis A, Decat P, Vega B, Cordova K, Temmerman M, Degomme O, Michielsen K: A cross-sectional study on attitudes toward gender equality, sexual behavior, positive sexual experiences, and communication about sex among sexually active and non-sexually active adolescents in Bolivia and Ecuador.Glob Health Action 2014;7:24089.

25. PAHO/CDC: Summary Report: Violence Against Women in Latin America and the Caribbean: A Comparative Analysis of Population-Based Data from 12 Countries. Washington, DC: Pan American Health Organization / Centers for Disease Control and Prevention; 2013.

26. Beckwith J: Knowledge, attitudes, and practices in reproductive and sexual health. McGill J Med 2006;9:119–125.

27. Goicolea I, Wulff M, Ohman A, San Sebastian M: Risk factors for pregnancy among adolescent girls in Ecuador’s Amazon basin: a case-control study.Rev Panam salud publica/Pan Am J public Heal. 2009;26:221–228.

28. Guijarro S, Naranjo J, Padilla M, Gutiérez R, Lammers C, Blum RW: Family risk factors associated with adolescent pregnancy: Study of a group of adolescent girls and their families in Ecuador. J Adolesc Heal. 1999;25:166–172.

29. Cabrero F, Minango G, Ávila M, Alexandra Costales SL, Autoridades, Allnutt DDC, Durán DXDX, Arellano DCL: Situación de Salud de Los Y Las Jóvenes Indígenas En Ecuador. Quito: Ministerio de Salud Pública; 2010.

30. Lundgren R, Amin A: Addressing intimate partner violence and sexual violence among adolescents: emerging evidence of effectiveness.J Adolesc Health 2015;56(1 Suppl):S42–50.

31. UNFPA Ecuador: Estudio de Percepción Sobre Planificación Familiar Y Uso de Métodos Anticonceptivos. Quito: UNFPA; 2011.

32. Cordova Pozo K, Chandra-Mouli V, Decat P, Nelson E, Meyer S De, Jaruseviciene L, Vega B, Segura Z, Auquilla N, Hagens A, Braeckel D Van, Michielsen K: Improving adolescent sexual and reproductive health in Latin America : reflections from an International Congress. Reproductive Health 2015; 12(1):1–7.

33. Goicolea I, Wulff M, Sebastian MS, Ohman A: Adolescent pregnancies and girls’ sexual and reproductive rights in the amazon basin of Ecuador: an analysis of providers' and policy makers' discourses.BMC Int Health Hum Rights 2010;10:12.

34. Borile M: Adolescencia: La necesidad de su abordaje integral. Presented at International Congress Promoting Sexual and Reproductive Health. Cuenca; 2014.

35. MSP: Política Nacional de Salud Y Derechos Sexuales Y Reproductivos [National Policy for Sexual and Reproductive Rights and Health]. Quito: Ministerio de Salud Público; 2005.

36. Naranjo Pinto J: Estrategia De Fortalecimiento Y Abogacia De Servicios Con Atencion Amigable Y / O Diferenciada Para Adolescentes. Quito: Ministerio de Salud Pública; 2012.

37. Dick B, Ferguson J, Chandra-Mouli V, Brabin L, Chatterjee S, Ross D: Review of the Evidence for Interventions to Increase Young People’s Use of Health Services in Developing Countries. Technical Report Series 938, 151. Geneva: WHO; 2006.

38. WHO: Making Health Services Adolescent Friendly - Developing National Quality Standards for Adolescent Friendly Health Services. Geneva: WHO; 2012.
